# Effects of Social Distancing on Quality of Life and Emotional-Affective Sphere of Caregivers and Older Patients Hospitalized in Rehabilitation Departments during COVID-19 Quarantine: An Observational Study

**DOI:** 10.3390/diagnostics12061299

**Published:** 2022-05-24

**Authors:** Irene Aprile, Francesca Falchini, Emiliano Mili, Alessia Mastrorosa, Emanuele Langone, Rita Mosca, Stefano Larocca, Michele Lategana, Loredana Aiello, Angela Lorusso, Mariacristina Siotto, Daniele Giansanti, Marco Germanotta

**Affiliations:** 1 IRCCS Fondazione Don Carlo Gnocchi, 50143 Florence, Italy; iaprile@dongnocchi.it (I.A.); ffalchini@dongnocchi.it (F.F.); emili@dongnocchi.it (E.M.); elangone@dongnocchi.it (E.L.); rmosca@dongnocchi.it (R.M.); slarocca@dongnocchi.it (S.L.); mlategana@dongnocchi.it (M.L.); laiello@dongnocchi.it (L.A.); alorusso@dongnocchi.it (A.L.); msiotto@dongnocchi.it (M.S.); 2 P.O.A. Cardarelli, S.C. Neurologia—Stroke Unit, 86100 Campobasso, Italy; alessiamst@gmail.com; 3 Centro Tisp, Istituto Superiore di Sanità, 00161 Roma, Italy; daniele.giansanti@iss.it

**Keywords:** COVID-19, rehabilitation, social distancing, anxiety, depression, quality of life

## Abstract

During the COVID-19 emergency, institutional social distancing conditions were established, preventing family and caregivers’ access to rehabilitation departments. Our study goal was to assess inpatients’ and caregivers’ anxiety, depression, and Quality of Life (QoL) during the Italian lockdown due to the pandemic. We investigated anxiety, depression, and QoL in 53 patients and 51 caregivers, using the Beck Anxiety Inventory (BAI), the Beck Depression Inventory-II (BDI-II), and the Short Form 36 Health Survey (SF36). These questionnaires were given to patients after one (T0) and two months (T1) since the hospitalization. The BAI showed that anxiety was moderate for 7.5% of patients and 23.5% of caregivers, and severe for 35.8% of patients and 17.6% of caregivers. The BDI found moderate depression in 11.3% of patients and 15.7% of caregivers, and severe depression in 34.0% of patients and 9.8% of caregivers. Depression was higher in patients than caregivers, while no differences were detected in anxiety. Compared to normative data, patients’ QoL declined in all eight SF36 dimensions, while caregivers’ QoL declined only in social, emotional, and mental components. Unexpectedly, patients still hospitalized at T1 showed significant improvements in both anxiety and three QoL subscores. These findings emphasize the importance of psychological support for patients and their families.

## 1. Introduction

On 11 February 2020, the WHO announced the name of the respiratory disease caused by the new coronavirus: COVID-19. This novel coronavirus disease, caused by the virus SARS-CoV-2, has spread worldwide, with 513,955,910 cases and 6,249,700 deaths in about 230 countries (WHO update on 6 May 2022). Since the onset of the COVID-19 outbreak, and its subsequent worldwide diffusion, Italy has been among the first affected countries, and the number of recorded cases is among the highest in the world: after 2 years from the beginning of the pandemic, 16,682,626 confirmed cases and 164,179 deaths (Italian Ministry of Health, update on 6 May 2022) were reported. On 9 March 2020, in response to the country’s growth of the COVID-19 pandemic, the government of Italy decreed urgent measures, promoting social distancing to limit the spread of the virus. Since 11 March, any essential work activities or recreational activities were suspended or converted to the so-called “smart-working”. Citizens were forced to stay at home and journeys were drastically reduced; the freedom to leave home was limited to urgent health circumstances or for healthcare workers. The Italian population has never been subjected to such a lockdown.

To minimize the number of new cases, most nations, including Italy, used social distancing as their primary strategy [1]. The most adverse consequences of social distancing included loneliness, disruption of routine activities, restriction of freedom of movement, reduction in employment and income, and a shortage of medical services for disorders other than COVID-19 [2,3,4].

One of the most concerning characteristics of the COVID-19 pandemic is the involvement of frail and vulnerable persons, particularly the elderly, with many comorbidities or chronic conditions, and those with disabilities [5]. Indeed, rehabilitation services and activities were halted, and only post-acute and subacute rehabilitation programs in hospitals and healthcare institutions, respectively, were available for patients who were unable to return home following an acute occurrence.

The COVID-19 emergency led to the establishment of institutional social distancing conditions; thus, access for family members and/or caregivers to rehabilitation departments was prohibited. This measure, although necessary, caused patients and family members to feel uneasy for the first time.

Patients admitted to our facilities for rehabilitation treatment could not receive visits from their relatives or caregivers for the entire rehabilitation program, given that rehabilitation intervention can last for months. Similarly, also, in older persons’ residential facilities (e.g., nursing homes), visits were blocked. These measures led patients to become increasingly lonely over time as their already limited social opportunities were further reduced [6].

To overcome the distress, “technological” solutions, such as video calls, were used in order to maintain communication between patients and family members. Despite this, both patients and family members/caregivers are likely to be affected by the COVID-19 emergency and the constraints that come with social distance.

Indeed, people with disabilities are more likely to suffer from mood disorders, including anxiety and depression. Moreover, fragile patients require polymedications or specific drugs that can be associated with neuropsychological side effects [7,8]. 

It has been demonstrated that adults with impairments are more likely to experience more psychosocial stressors than persons without disabilities [9]. Depression, especially in patients after stroke, is a relevant, additional disabling factor, which is responsible for ~15% of the increased disability [10].

More evidence has shown that the COVID-19 pandemic led to psychological issues not only in patients but also in health care workers with an enhancement of depression, anxiety, and Post-Traumatic Stress Disorder [11]. Anxiety and depression levels in our patients and their relatives might have been negatively influenced by social restrictions, including the impossibility to see the relatives, as well as the fear of getting severely sick. Furthermore, it has been shown that psychological distress does not arise just as a result of fear of contracting COVID-19; various other variables also play a significant role. [12,13,14].

The goal of our study was, therefore, to look at the levels of anxiety, depression, and quality of life (QoL) in a group of inpatients and their relatives during the Italian lockdown due to the SARS-CoV-2 pandemic. Specifically, we sought to investigate (a) whether the social distance between patients undergoing rehabilitation for orthopedic or neurological disabilities and their relatives/caregivers impacted the emotional-affective sphere and the QoL and (b) the evolution of anxiety, depression, and QoL over time.

## 2. Materials and Methods

This is an observational study conducted in four centers of the Fondazione Don Carlo Gnocchi ONLUS, in Italy. It is the largest private no-profit organization in the field of rehabilitation in Italy. The study was approved by our Ethics Committee on 16 April 2020 (FDG_16.4.2020) and registered on clinicaltrials.gov (NCT04408196).

### 2.1. Participants and Intervention

We enrolled patients admitted to our rehabilitation facilities (inpatient setting) because of neurological or orthopedic diseases, or to our nursing home, along with their caregivers. Patients and caregivers were unable to see each other because of the limitation imposed by the pandemic. We included patients aged 65 years or older, hospitalized for at least a month, while we excluded patients and caregivers with a score on the Mini-Mental State Examination (MMSE) scale lower than 24.

During hospitalization, patients underwent a daily 60 min physical therapy rehabilitation intervention, six times per week. Patients were provided with neuromotor, orthopedic, pulmonary, cognitive, or occupational treatments, according to their needs. Since social distancing measures prevented the use of rehabilitation gyms, the treatments were administered in the patient’s room. In addition, when only routine clinical visits were required, patient care included three visits each day by a nurse and one visit every two to three days by a physician. Finally, every two days, a psychologist assisted patients in making videophone calls to their families.

### 2.2. Instruments

Demographic (for patients and caregivers) and anamnestic (only for patients) data were collected. Moreover, three questionnaires were administered to patients and caregivers:

- The Short Form (36) Health Survey (SF36), to evaluate the QoL [15];
- The Beck Anxiety Inventory (BAI), to evaluate the emotional state [16];
- The Beck Depression Inventory-II (BDI-II), to evaluate mood [17];

Finally, disability was assessed in patients using the modified Barthel Index (mBI) [18].

### 2.3. Procedure

The aforementioned questionnaires were administered after one month of hospitalization (T0), and after two months (T1). The questionnaires were provided to patients by a researcher involved in the study, while the caregivers were given self-administered questionnaires developed on Microsoft Forms. This form also allowed us to obtain consent for the use of data.

### 2.4. Statistical Analysis

Descriptive statistics were used for the evaluation of demographic data of patients and caregivers. Owing to the ordinal nature of the questionnaire, non-parametric tests were used. Anxiety and depression at T0, as evaluated by the selected questionnaire, were compared between patients and caregivers by means of the Mann–Whitney U-test; moreover, SF-36 questionnaire data obtained at T0 from both patients and caregivers were compared with the normative values reported by Apolone et al. [15] using the Kruskal–Wallis test, with post hoc tests, when necessary.

The correlations between depression, anxiety, and QoL with demographic and clinical characteristics were investigated using the Spearman rank correlation coefficient.

Finally, to assess any changes in depression, anxiety, and QoL, data obtained at two timepoints (T0 and T1) in patients, were compared by means of the Wilcoxon signed-rank test, considering hospitalized and discharged patients separately.

SPSS software package (IBM SPSS Statistics for Windows, Version 25.0. Armonk, NY, USA: IBM Corp.) was used for statistical analyses, and a *p* value lower than 0.05 was deemed significant.

## 3. Results

In this study, 53 patients (31 women, 22 men) and 51 caregivers (33 women, 18 men), were enrolled in five centers of the Fondazione Don Carlo Gnocchi (Roma, Sant’Angelo dei Lombardi, Acerenza, and Tricarico).

**Figure 1:**
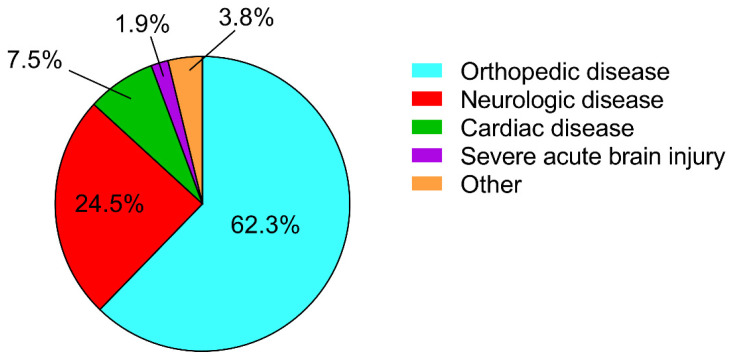
Patients’ diagnosis.

The patients’ mean age was 75 ± 7 years, (range 65–91). Patients’ disability, as measured by the mBI, was 52 ± 22 (range 3–94), while the mean score of the MMSE was 26.9 ± 2.0 (range 24–30). As reported in Figure 1, about 60% of the patients were admitted to our rehabilitation department because of orthopedic disease.

**Figure 2:**
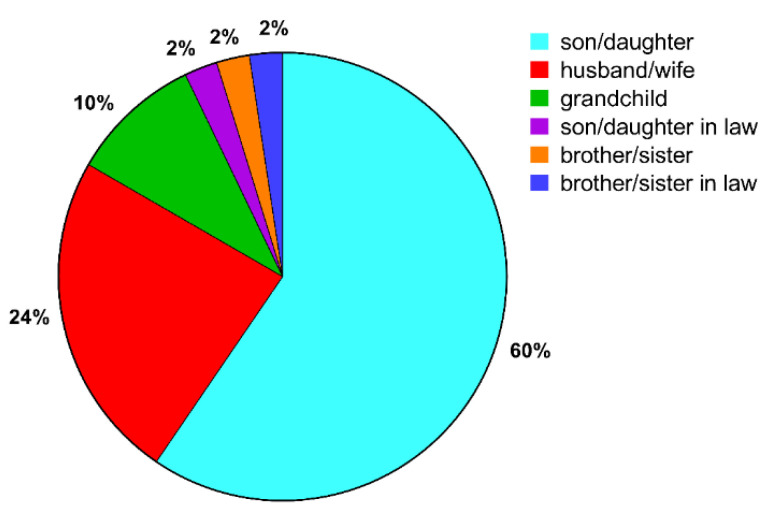
Relationship status caregiver-patient.

With respect to the caregivers, their mean age was 51 ± 13 years (range 23–81): as reported in Figure 2, about 60% were a son/daughter and, notably, all of them were a patient’s relative.

**Figure 3:**
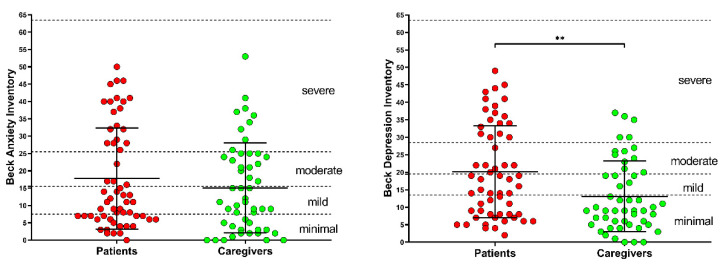
Beck Anxiety Inventory scores and Beck Depression Inventory scores in patients and caregivers. The asterisks indicate a statistically significant difference (** *p* < 0.01, according to the Mann–Whitney U test).

In Figure 3, BAI and BDI values for patients and caregivers, together with their mean values, are reported. According to the BAI scores, anxiety was minimal (0 ≤ BAI ≤ 7) for 32.1% of patients and 33.3% of caregivers; mild (8 ≤ BAI ≤ 15) for 24.5% of patients and 25.5% of caregivers; moderate (16 ≤ BAI ≤ 25) for 7.5% of patients and 23.5% of caregivers; and finally, severe (26 ≤ BAI ≤ 63) for 35.8% of patients and 17.6% of caregivers, with no difference between patients and caregivers (*p* = 0.156).

With respect to the depression level, as measured by the BDI, it was minimal (0 ≤ BDI ≤ 13) for 34.0% of patients and 64.7% of caregivers; mild (14 ≤ BDI ≤ 19) for 20.8% of patients and 9.8% of caregivers; moderate (20 ≤ BDI ≤ 28) for 11.3% of patients and 15.7% of caregivers; and finally, severe (29 ≤ BDI ≤ 63) for 34.0% of patients and 9.8% of caregivers. According to the statistical analysis, it was higher in patients, when compared to caregivers (*p* = 0.002).

**Figure 4:**
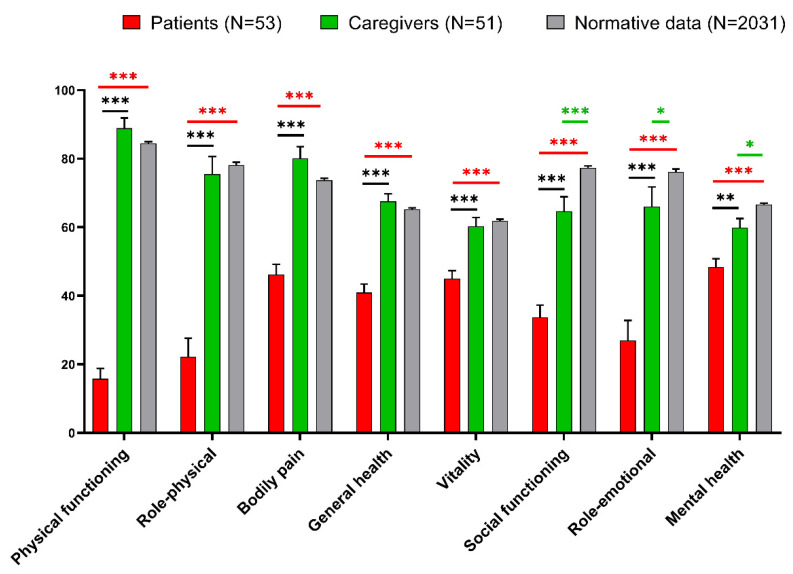
36-Item Short-Form Health Survey (SF36) data from patients and caregivers, together with normative Italian data. The asterisks indicate a statistically significant difference between groups, according to the post hoc analysis (* *p* < 0.05, ** *p* < 0.01, *** *p* < 0.001). Red asterisks refer to the comparison patients-normative data, green asterisks refer to the comparison caregivers-normative data, and black asterisks refer to the comparison patients-caregivers.

Concerning the SF-36 questionnaire results, the eight items produced by the responses were compared between patients, caregivers, and normative data from an Italian population (n = 2031), according to Apolone et al. [15], and reported in Figure 4.

As expected, patients’ QoL significantly decreased when compared to caregivers and normative data, as seen by statistically significant reductions in all SF-36 subscores. Unexpectedly, when compared to normative data, caregivers also demonstrated a statistically significant deterioration in social functioning, role-emotional, and mental health dimensions.

The correlations between anxiety, depression, and QoL with age, disability, and cognitive function in patients are reported in Table 1. We found that anxiety (BAI scores) and depression (BDI scores) were correlated with cognitive functions (MMSE scores). With respect to the QoL domains, we found that physical functioning was inversely correlated with patients’ age and disability, while the SF-36 subscores, general health, vitality, social functioning, and mental health, were inversely correlated with cognitive functions (MMSE scores).

**Figure 5:**
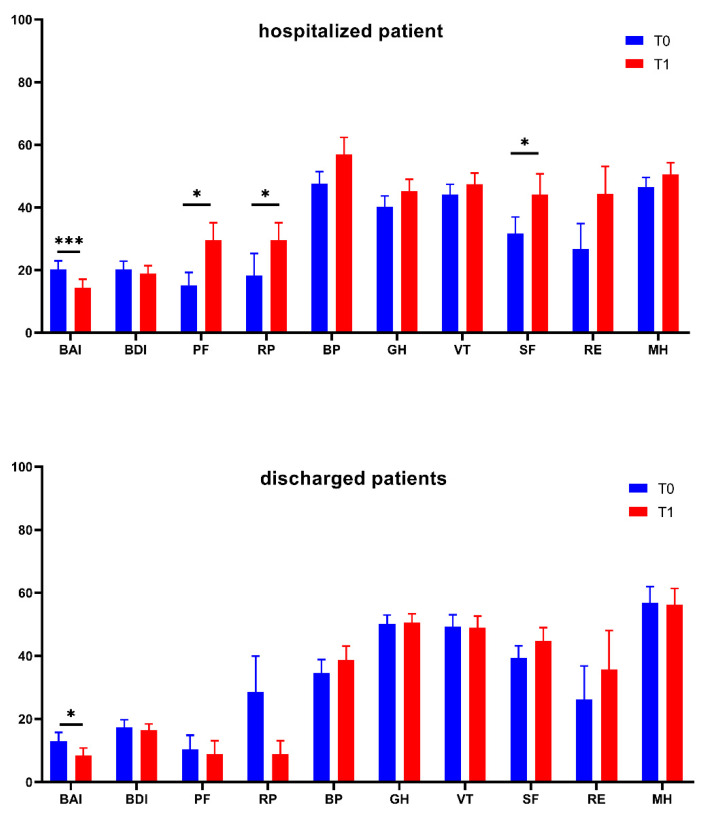
Evolution of anxiety, depression, and Quality of Life in patients between T0 (at least 1 month after the hospitalization) and T1 (one month after T1). BAI: Beck Anxiety Inventory; BDI: Beck Depression Inventory; PF: SF36—Physical Functioning; RP: SF36—Role-functioning Physical; BP: SF36—Bodily Pain; GH: SF36—General Health; VT: SF36—Vitality; SF: SF36—Social Functioning; RE: SF36—Role-functioning Emotional; MH: SF36—Mental Health. The asterisks indicate a statistically significant difference (* *p* < 0.05; *** *p* < 0.001).

Finally, the evolution of anxiety, depression, and QoL in patients one month after the first evaluation is reported in Figure 5. In the analysis, patients who were still in the hospital and those who had been discharged were treated separately. In both, we observed a statistically significant reduction in anxiety; with respect to QoL, after a month, hospitalized patients improved in three out of eight QoL domains (physical functioning, role-functioning physical, and social functioning), while discharged patients did not improve their QoL.

## 4. Discussion

Due to the lockdown enforced by COVID-19, patients admitted for rehabilitation in healthcare facilities endured isolation and were not able to be visited by their loved ones. In this study, we expected that imposed isolation of patients could have a negative impact on their quality of life and emotional sphere. In fact, it has been shown that there is a significant relationship between the experience of COVID-19, social distancing, and mental health [19]. More uncertainty concerned the reaction that caregivers would have manifested because of the inability to visit their hospitalized relatives.

To mitigate the impacts of isolation, measures (such as video calls) were put in place to maintain contact between the patients and their families. However, this solution, forced by necessity, is not equivalent to in-person visits by family members [20].

Regarding the emotional-affective sphere, as expected, patients, showed signs of depression, which is common in frail patients with orthopedic and neurological illnesses who require rehabilitation due to their transient or, in some more severe cases, permanent impairment [21]. Our study evidenced a surprising finding on anxiety levels. In fact, we expected that social distancing could also cause a state of mild anxiety in family members, but we were not expecting caregivers’ anxiety to be comparable to that of patients. We believe that both the distance from the loved ones, as well as the overall fear caused by the epidemic, are factors that can be related to the reported significant level of anxiousness in caregivers, as claimed in another study [22]. In terms of quality of life, patients had a deterioration in all eight SF36 dimensions, while caregivers had lower scores in the social, emotional, and mental components of QoL when compared to normative data. This evidence is noteworthy and we feel it is due to the unique circumstances in which the relatives found themselves: the inability to see their loved ones while also dealing with a pandemic. Moreover, patients who are older or have severe disabilities reported a reduced QoL in both physical and mental aspects. This conclusion is supported by a few studies [23,24]. Moreover, these findings are consistent with other research that suggests that a higher percentage of people feel emotional distress following unanticipated terrifying incidents and catastrophic disasters [25,26] and that there is a close relationship between the COVID-19 pandemic, social distancing, and mental health [19].

Note that the depression and anxiety levels are unrelated to age, or disability. In fact, in our sample, depression and anxiety, while evident, were not correlated to the modified Barthel Index. This result is surprising, since usually, individuals with disabilities who receive rehabilitation treatment have a low mood, which is greater, the more severe the disability [27,28]. With respect to QoL, we found that only one out of eight QoL subscores (i.e., the Physical Function subscore) was correlated with disability, while no mental subscores were correlated to the mBI. This finding suggests that fear of COVID and social alienation are more likely causes of emotional impairment and deterioration in the mental aspect of QoL. Of course, ours is a hypothesis since we cannot measure separately the impact of disability and COVID-19 on patients’ anxiety, depression, and QoL and this can be considered a limitation of the study. However, the lack of correlation with disability, as measured by the modified Barthel Index, leads us to hypothesize a strong impact of COVID-19 on patients’ anxiety, depression, and QoL.

The conclusion concerning QoL and the emotional sphere in patients after one month from the initial evaluation was undoubtedly the most unexpected. Indeed, we assumed discharged patients to be in better condition, but they only show a reduction in anxiety (which is to be expected given that they have returned to their families and routines). As a matter of fact, patients who were still hospitalized one month after the initial evaluation showed considerable improvements not only in anxiety, but also in some QoL subscores (physical functioning, role-functioning physical, and social functioning subscores). This finding led us to believe that patients felt safer in rehabilitation centers where, although being separated from their loved ones, they were kept in a tightly regulated environment to prevent the COVID infection.

These findings highlight the significance of psychological support for both patients and their families, as well as technology solutions that can help minimize the effects of difficult-to-maintain estrangements, such as that between patients and their loved ones.

The lockdown has had an impact on the whole community, especially in terms of separation from loved ones. This was particularly difficult for those who were unable to spend the lockdown period with their family. This is reflected in the fact that not only patients but even caregivers have been affected, reporting high levels of anxiety and a decreased quality of life. Indeed, as a result of the preventive precautions put in place during the pandemic, family caregivers have been burdened with additional problems, prompting the creation of support and relief programs tailored to this demographic group [29].

While we have survived the most challenging aspects of the pandemic (such as the lockdown), these facts should serve as a helpful guide to ensure that similar problems do not arise in the future.

One way to be prepared is to improve technological systems that can reduce distances (e.g., a video-presence system in all patient rooms with easy access even for older patients), as well as to raise awareness of the consequences and the necessity of adequate psychological support for patients and their families.

## Figures and Tables

**Table 1:** Correlations between anxiety, depression, and Quality of Life with age, disability, and cognitive function. mBI: Modified Barthel Index; MMSE: Mini-Mental State Examination; SF36: Short Form 36; BAI: Beck anxiety inventory; BDI: Beck depression inventory; PF: Physical Functioning; RP: Role-functioning Physical; BP: Bodily Pain; GH: General Health; VT: Vitality; SF: Social Functioning; RE: Role-functioning Emotional; MH: Mental Health. Values in bold indicate statistically significant correlations (* *p* < 0.05; ** *p* < 0.01).

	Age		mBI		MMSE	
	r	*p*	r	*p*	r	*p*
BAI	0.199	0.154	−0.149	0.286	0.376 **	**0.006**
BDI	0.219	0.116	0.286	0.482	0.347 *	**0.011**
SF36-PF	−0.364 **	**0.007**	0.449 **	**0.001**	0.071	0.613
SF36-RP	−0.198	0.155	0.173	0.215	−0.074	0.598
SF36-BP	−0.084	0.548	0.147	0.294	0.029	0.837
SF36-GH	−0.175	0.209	0.131	0.350	−0.290 *	**0.035**
SF36-VT	−0.184	0.187	0.119	0.397	−0.351 **	**0.010**
SF36-SF	−0.181	0.196	0.103	0.464	−0.327 *	**0.017**
SF36-RE	−0.202	0.147	0.055	0.696	−0.200	0.151
SF36-MH	−0.198	0.156	0.257	0.063	−0.292 *	**0.034**

## Data Availability

The data that support the findings of this study are available from the corresponding author upon reasonable request.
